# Unique somato-dendritic distribution pattern of Kv4.2 channels on hippocampal CA1 pyramidal cells

**DOI:** 10.1111/j.1460-9568.2011.07907.x

**Published:** 2012-01

**Authors:** Katalin Kerti, Andrea Lorincz, Zoltan Nusser

**Affiliations:** Laboratory of Cellular Neurophysiology, Institute of Experimental Medicine, Hungarian Academy of SciencesBudapest, Hungary

**Keywords:** dendrite, hippocampus, immunohistochemistry, potassium channels, pyramidal cell, rat

## Abstract

A-type K^+^ current (I_A_) plays a critical role in controlling the excitability of pyramidal cell (PC) dendrites. *In vitro* dendritic patch-pipette recordings have demonstrated a prominent, sixfold increase in I_A_ density along the main apical dendrites of rat hippocampal CA1 PCs. In these cells, I_A_ is mediated by Kv4.2 subunits, whose precise subcellular distribution and densities in small-diameter oblique dendrites and dendritic spines are still unknown. Here we examined the densities of the Kv4.2 subunit in 13 axo-somato-dendritic compartments of CA1 PCs using a highly sensitive, high-resolution quantitative immunogold localization method (sodium dodecyl sulphate-digested freeze-fracture replica-labelling). Only an approximately 70% increase in Kv4.2 immunogold density was observed along the proximo-distal axis of main apical dendrites in the stratum radiatum with a slight decrease in density in stratum lacunosum-moleculare. A similar pattern was detected for all dendritic compartments, including main apical dendrites, small-diameter oblique dendrites and dendritic spines. The specificity of the somato-dendritic labelling was confirmed in Kv4.2^−/−^ tissue. No specific immunolabelling for the Kv4.2 subunit was found in SNAP-25-containing presynaptic axons. Our results demonstrate a novel distribution pattern of a voltage-gated ion channel along the somato-dendritic surface of CA1 PCs, and suggest that the increase in the I_A_ along the proximo-distal axis of PC dendrites cannot be solely explained by a corresponding increase in Kv4.2 channel number.

## Introduction

Understanding the way nerve cells integrate their synaptic inputs requires the knowledge of the precise subcellular location and densities of voltage-gated ion channels (e.g. Na^+^, Ca^2+^, K^+^, mixed cation) on the axo-somato-dendritic surface of the cells. Among the voltage-gated ion channels, K^+^ channels (Kv) are molecularly the most diverse and play a powerful role in controlling neuronal excitability (Hille, 2001; Gutman *et al.*, 2003). Particular attention has been paid to the rapidly inactivating or A-type K^+^ current (I_A_) due to their widespread distribution in the CNS (Serodio & Rudy, 1998) and their low activation threshold that is around the resting membrane potential of most neurons (Birnbaum *et al.*, 2004). It has been elegantly demonstrated that I_A_ has a major role in a large variety of dendritic processes, including the control of local Na^+^ spike initiation and propagation (Losonczy *et al.*, 2008), the backpropagation of axonally generated action potentials into the dendrites (Hoffman *et al.*, 1997; Migliore *et al.*, 1999), synaptic integration and plasticity (Margulis & Tang, 1998; Cash & Yuste, 1999; Watanabe *et al.*, 2002; Frick *et al.*, 2004; Kim & Hoffman, 2008; Makara *et al.*, 2009). To fulfil all these functional roles, I_A_ must be present in the dendrites of pyramidal cells (PCs). Direct dendritic patch-clamp recordings have provided evidence for its presence in CA1 PC dendrites, and revealed a sixfold increase in its density as a function of distance from proximal to distal main apical dendrites (Hoffman *et al.*, 1997).

Using Kv4.2^−/−^ mice, Chen *et al.* (2006) have demonstrated that in hippocampal CA1 PCs dendritic I_A_ is exclusively mediated by the Kv4.2 subunit. This is consistent with the results of light microscopic immunohistochemical studies revealing strong Kv4.2 immunosignal in the dendritic layers of the CA1 area (Maletic-Savatic *et al.*, 1995; Varga *et al.*, 2000; Rhodes *et al.*, 2004; Jinno *et al.*, 2005). However, it has been noticed that the intensity of the immunoperoxidase or fluorescent signal is rather uniform across the stratum radiatum (SR), with a slight decrease in the stratum lacunosum-moleculare (SLM). One possible explanation for the discrepancy between the density of I_A_ and that of Kv4.2 immunolabelling is that the oblique dendrites or dendritic spines or axon terminals contain a high density of Kv4.2 subunit, which are superimposed and therefore mask the increasing, but lower, density of Kv4.2 subunit in the main apical dendrites.

Here we aimed to determine whether the known increase in I_A_ density in main apical dendrites of CA1 PC is mirrored by a corresponding increase in the density of Kv4.2 subunit, and to quantitatively compare the density of the Kv4.2 subunit in large- and small-diameter dendritic and axonal compartments. To achieve our aims, we carried out immunogold localization of the Kv4.2 subunit with a highly sensitive, quantitative, electron microscopic immunogold localization technique [sodium dodecyl sulphate-digested freeze-fracture replica-labelling (SDS-FRL); Fujimoto, 1995; Rash *et al.*, 1998; Masugi-Tokita & Shigemoto, 2007].

## Materials and methods

All procedures were carried out in accordance with the ethical guidelines of the Institute of Experimental Medicine of the Hungarian Academy of Sciences, which is in line with the European Union regulation of animal experimentations.

### Tissue preparation

Seven adult male Wistar rats (P25–P41; bred in the Institute of Experimental Medicine’s Animal Facility), three male wild-type and three Kv4.2^−/−^ mice (129/SvEv background; Chen *et al.*, 2006; P68–P217; kindly provided by Prof. Daniel Johnston from the University of Texas, Austin, USA) were deeply anaesthetized with ketamine (0.5 mL/100 g) and then were transcardially perfused with ice-cold fixative. For immunofluorescent reactions animals were perfused with a fixative containing either 2 or 4% paraformaldehyde and 15v/v% picric acid made up in 0.1 m phosphate buffer (PB) for 20 min. Afterwards, 60 μm coronal forebrain sections were cut with a vibratome (VT1000S; Leica Microsystems, Wetzlar, Germany). For SDS-FRL, animals were perfused with a fixative containing 2% paraformaldehyde and 15v/v% picric acid in 0.1 m PB for 16 min. Coronal sections of 80 μm thickness were cut from the dorsal hippocampus with a vibratome and were cryoprotected in 30% glycerol.

### Immunofluorescent reactions

Free-floating sections were blocked in 10% normal goat serum (NGS), followed by an incubation in the solution of rabbit anti-Kv4.2 (1 : 500; APC-023; Alomone Labs, Jerusalem, Israel) antibody made up in Tris-buffered saline (TBS) containing 2% NGS. Sections were then incubated in Alexa488- or Cy3-conjugated goat anti-rabbit IgGs made up in TBS containing 2% NGS for 2 h. Images from the CA1 region were acquired using a confocal laser-scanning microscope (FV1000; Olympus, Tokyo, Japan) with a 20 × objective (UPLANSAPO, UIS2, NA = 0.75).

### SDS-FRL

Small blocks from the CA1 region were frozen in a high-pressure freezing machine (HPM 100, Leica Microsystem) and fractured at −135 °C in a freeze-fracture machine (BAF060, Leica Microsystem), as described previously (Masugi-Tokita *et al.*, 2007; Lorincz & Nusser, 2010). The fractured tissue surfaces were coated with thin layers of carbon (5 nm), platinum (2 nm) and carbon (20 nm). Tissue debris from the replicas was digested in a solution containing 2.5% SDS and 20% sucrose in TBS (pH 8.3) at 80 °C overnight. Following several washes in TBS containing 0.05% bovine serum albumin (BSA), replicas were blocked in TBS containing 0.1% BSA for 1 h, then incubated overnight in blocking solution containing the following primary antibodies: rabbit anti-Kv4.2 (1 : 5000; Alomone Labs); mouse anti-postsynaptic density (PSD)-95 (1 : 3000; MAB1598; Chemicon, Billerica, MA, USA); mouse anti-γ-aminobutyric acid (GABA)_A_R β3 (1 : 800; 75–149; NeuroMab); mouse anti-SNAP-25 (1 : 5000; 111 001; SYSY; Goettingen, Germany). Replicas were then incubated for 2 h in TBS containing 1% BSA and the following secondary antibodies: goat anti-mouse IgGs coupled to 5-nm or 15-nm gold particles (1 : 100; British Biocell International, Cardiff, UK); or goat anti-rabbit IgGs coupled to 10-nm gold particles (1 : 100; British Biocell International). Finally, replicas were rinsed in TBS and distilled water before they were picked up on copper parallel bar grids. Specimens were analysed with a transmission electron microscope (JEM-1011, JEOL, Tokyo, Japan) equipped with a high-resolution bottom-mounted CCD camera (Cantega G2, Olympus Soft Imaging Solution GmbH, Munster, Germany).

### Quantitative analysis of immunogold labelling for the Kv4.2 subunit

Quantitative analysis of CA1 PC somata and 11 dendritic compartments was performed only on complete replicas where the stratum pyramidale (SP), SR and SLM could be clearly distinguished (*n* = 5 rats). The subcellular compartments were imaged with a Cantega G2 camera at 10 000–12 000 × magnification, and were grouped based on their calculated distance from the SP. The distance of an individual process from the SP was calculated from the position of the SP and from the stage coordinate (*X*, *Y* ) of the process using the following equation:


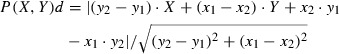


The position of the SP was determined by an imaginary line connecting two end points of the SP with the coordinates *x*_1_, *y*_1_ and *x*_2_, *y*_2_. According to this layers were categorized as follows: 0–120 μm: proximal SR; 120–240 μm: middle SR; 240–360 μm: distal SR; and above 360 μm: SLM. The main apical dendrites, oblique dendrites and dendritic spines were grouped according to these criteria. Oblique dendrites were identified based on their small diameter and the presence of at least one emerging spine from the dendritic shaft. Spines were identified based on either their ultrastructure (e.g. small-diameter structure emerging from a dendrite; *n* = 3 rats) or from the presence of a PSD (identified in double-labelling reactions with PSD-95) on isolated spine heads (*n* = 2 rats). The density of immunogold particles for the Kv4.2 subunit in dendritic spines was not significantly different (*P* > 0.05, *t*-test) between these two groups, therefore they were pooled together. The numbers of analysed profiles and counted gold particles are provided in Tables 1–3. The GABA_A_ receptor β3 subunit was used to identify GABAergic synapses on PC somata and dendrites. The immunolabelling for the Kv4.2 subunit was also quantified on axon terminals identified by a high density of gold particles labelling the SNAP-25 in three rats. Statistical comparisons were performed with the Statistica 8 Software (Scientific Computing, Rockaway, NJ, USA).

**Table 1:** Densities of gold particles labelling the Kv4.2 subunit in distinct somato-dendritic compartments of rat CA1 PCs

RAT	BG	Soma	Prox Ap Dend	Mid Ap Dend	Dist Ap Dend	Prox Ob Dend	Mid Ob Dend	Dist Ob Dend	Prox Sp	Mid Sp	Dist Sp	SLM Dend	SLM Sp
#1	1.1	8.2 (10, 371)	9.9 (9, 323)	11.6 (10, 394)	11.4 (10, 328)	15.3 (7, 99)	11.0 (12, 172)	14.2 (10, 175)	13.7 (14, 27)	18.7 (24, 87)	13.5 (12, 39)	9.6 (12, 202)	12.5 (16, 59)
#2	0.6	7.8 (10, 352)	13.4 (10, 352)	17.4 (10, 442)	15.6 (11, 367)	16.3 (10, 191)	17.8 (8, 239)	18.3 (13, 292)	19.7 (12, 41)	17.3 (13, 32)	17.3 (15, 50)	8.7 (19, 327)	8.8 (7, 15)
#3	1.7	8.5 (10, 463)	6.5 (11, 324)	12.1 (9, 300)	11.0 (10, 256)	11.2 (10, 124)	15.9 (10, 183)	18.6 (10, 200)	9.0 (8, 7)	15.7 (12, 22)	16.9 (7, 10)	14.3 (16, 395)	20.4 (6, 16)
#4	0.2	5.4 (8, 163)	5.0 (9, 240)	11.6 (8, 296)	11.3 (8, 238)	7.6 (8, 83)	8.5 (8, 64)	7.7 (9, 78)	8.8 (12, 16)	14.2 (10, 19)	10.6 (11, 12)	5.7 (8, 98)	7.6 (5, 8)
#5	0.1	4.8 (8, 185)	4.1 (7, 140)	6.9 (10, 284)	7.7 (9, 241)	4.6 (5, 27)	7.4 (10, 116)	7.6 (10, 96)	3.6 (96, 3)	9.5 (11, 20)	9.6 (12, 15)	6.2 (9, 178)	6.5 (13, 16)
Mean	**0.7**	**6.9** (9, 307)	**7.8** (9, 276)	**11.9** (9, 343)	**11.4** (10, 286)	**11.0** (8, 105)	**12.1** (10, 155)	**13.3** (10, 168)	**11.0** (10, 19)	**15.1** (14, 36)	**13.6** (11, 25)	**8.9** (13, 240)	**11.2** (9, 23)
SD	0.7	1.7 (1, 128)	3.8 (1, 87)	3.7 (1, 71)	2.8 (1, 58)	5.0 (2, 60)	4.6 (2,67)	5.4 (2, 86)	6.1 (3, 15)	3.5 (6, 29)	3.5 (3, 18)	3.4 (5, 119)	5.7 (5, 21)

Density values are provided in gold/μm^2^. In parenthesis, the first number indicates the number of analyzed profiles and the second number denotes the number of counted gold particles.

**Table 2:** Densities of gold particles labelling the Kv4.2 subunit in axon terminals of rat, Kv4.2^+/+^ and Kv4.2^−/−^ mice CA1 PCs

Rat	BG	Mid Ap Dend	Axon	Mouse	BG	Mid Ap Dend	Axon	Mouse	BG	Mid Ap Dend	Axon
#1	0.14	7.0 (10, 349)	1.7 (84, 39)	Kv4.2^+/+^ #1	0.50	6.3 (10, 157)	0.9 (91, 20)	Kv4.2^−/−^ #1	0.80	1.2 (10, 30)	2.7 (93, 65)
#2	0.95	16.6 (7, 220)	2.8 (66, 62)	Kv4.2^+/+^ #2	0.50	11.3 (9, 303)	4.0 (67, 64)	Kv4.2^−/−^ #2	0.48	0.3 (7, 5)	1.8 (137, 66)
#3	0.40	9.2 (5, 169)	2.6 (39, 44)	Kv4.2^+/+^ #3	0.52	8.1 (7, 98)	3.0 (169, 110)	Kv4.2^−/−^ #3	0.48	1.9 (8, 34)	2.1 (98, 50)
Mean	0.50	11.0 (7, 246)	2.4 (63, 48)		0.50	8.6 (9, 186)	2.6 (109, 65)		0.59	1.2 (8, 23)	2.2 (109, 60)
SD	0.42	5.1 (3, 93)	0.6 (23, 12)		0.01	2.6 (2, 106)	1.6 (53, 45)		0.19	0.8 (2, 16)	0.4 (24, 9)

Density values are provided in gold/μm^2^. In parentheses, the first number indicates the number of analysed profiles and the second number denotes the number of counted gold particles.

**Table 3:** The immunoreaction is specific in all somato-dendritic subcellular compartments of CA1 PCs

Mouse	BG	Soma	Ap Dend	Ob Dend	SLM Dend	Spine
Kv4.2^−/−^ #1	0.37	0.42 (8, 13)	0.28 (26, 22)	0.47 (31, 11)	0.43 (9, 7)	0.18 (38, 1)
Kv4.2^−/−^ #2	0.41	0.54 (10, 27)	0.59 (21, 39)	0.45 (22, 10)	0.70 (11, 11)	1.22 (35, 5)
Kv4.2^−/−^ #3	0.29	1.31 (9, 54)	2.44 (25, 167)	3.29 (28, 82)	2.07 (12, 40)	3.34 (39, 16)
Mean	0.36	0.76 (9, 31)	1.10 (24, 76)	1.40 (27, 34)	1.07 (11, 19)	1.58 (37, 7)
SD	0.06	0.48 (1, 21)	1.17 (3, 79)	1.63 (5, 41)	0.88 (2, 18)	1.61 (2, 8)

Density values are provided in gold/μm^2^. In parentheses, the first number indicates the number of analysed profiles and the second number denotes the number of counted gold particles.

### Control experiments

Double-labelling experiments were included in the manuscript only if the measured background labelling on E-face and the measured specific P-face labelling for the Kv4.2 subunit on dendritic shafts was very similar to that obtained in single-labelling reactions. Furthermore, in the double-labelling experiments the lack of cross-reactivity of secondary antibodies was tested by omitting the primary antibody for the Kv4.2 subunit and checking the lack of 10-nm gold particles on the labelled structures (e.g. PSDs or in axon terminals for PSD-95 or SNAP-25, respectively). Lack of labelling demonstrates that the goat anti-rabbit secondary antibody does not recognize the mouse anti-SNAP-25 or PSD-95 primary antibodies. Non-specific background labelling was measured on E-face structures around the measured P-faces as described previously (Lorincz & Nusser, 2010).

To confirm the specificity of our Kv4.2 immunoreactions on SDS-digested replicas, the above-described immunogold reactions were repeated in three Kv4.2^+/+^ and three Kv4.2^−/−^ mice, with the exception that here the layer categorization was as follows: 0–100 μm: proximal SR; 100–200 μm: middle SR; 200–300 μm: distal SR; and above 300 μm: SLM. In Kv4.2^−/−^ mice the immunogold particle density for the Kv4.2 subunit measured on P-faces was similar to the gold particle density measured on E-faces in replicas obtained from wild-type mice and rats, validating our approach of estimating the level of non-specific labelling on E-faces.

## Results

### Distribution and specificity of the Kv4.2 subunit immunoreactivity in the hippocampal CA1 area

**Fig. 1:**
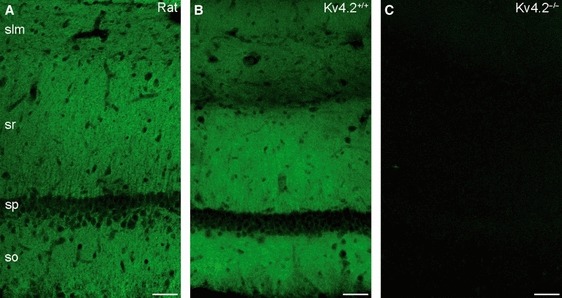
Distribution of the Kv4.2 subunit immunoreactivity in the hippocampus and the specificity of immunoreactions. (A) The immunfluorescent reaction shows strong, homogenous neuropil labelling for the Kv4.2 subunit in strata oriens (so) and radiatum (sr) of rat CA1 region, with a slight reduction of the immunolabelling in stratum lacunosum-moleculare (slm). (B) A very similar labelling pattern was observed in the mouse hippocampus. (C) The labelling was absent in the CA1 area of Kv4.2^−/−^ mice, demonstrating the specificity of the immunoreaction. sp, stratum pyramidale. All images are single confocal sections. Scale bars: 50 μm (A–C).

Light microscopic immunofluorescent reactions for the Kv4.2 subunit revealed a rather uniform labelling pattern throughout the stratum oriens and SR of the CA1 area of rat dorsal hippocampus with a slight reduction in the intensity in the SLM (Fig. 1A), in line with previous reports (Maletic-Savatic *et al.*, 1995; Varga *et al.*, 2000; Rhodes *et al.*, 2004; Jinno *et al.*, 2005). To validate the specificity of the immunofluorescent reactions, Kv4.2^−/−^ mice were used. The labelling pattern in control mice (Fig. 1B) was very similar to that obtained in rats. The complete lack of labelling in the Kv4.2^−/−^ mice (Fig. 1C) demonstrates that the immunolabelling at the light microscopic level is due to specific antibody–antigen interactions. To compare quantitatively the Kv4.2 subunit content of distinct somato-dendritic compartments, we carried out immunogold labelling using the SDS-FRL method. We systematically investigated gold particle densities in main apical dendrites, oblique dendrites and dendritic spines in the inner, middle and outer thirds of the SR, dendritic shafts and spines in the SLM in addition to the somata of CA1 PCs.

### High-resolution immunogold localization of the Kv4.2 subunit along the CA1 PCs somato-dendritic axis

**Fig. 2:**
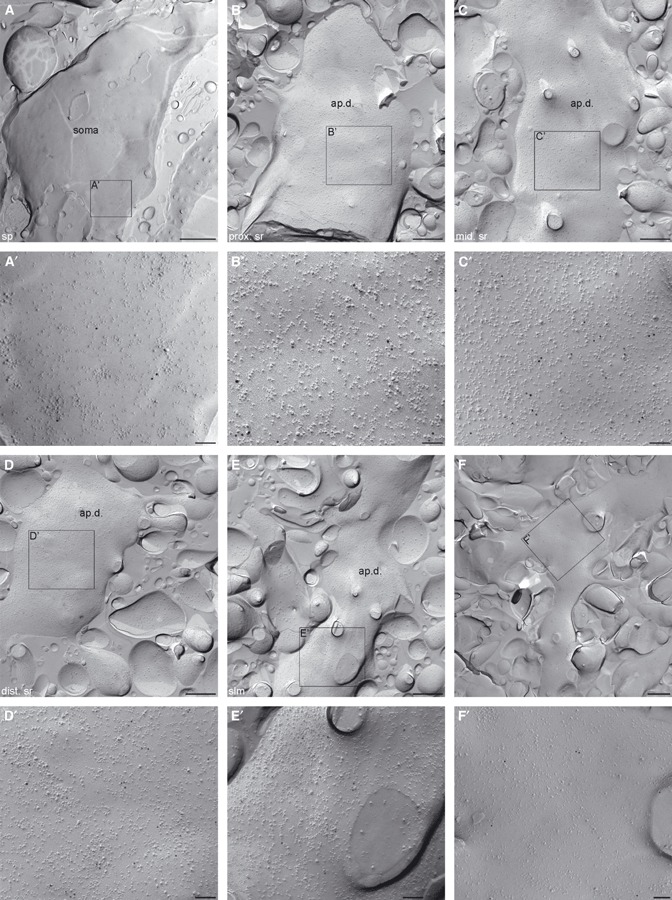
High-resolution immunogold localization of the Kv4.2 subunit in somata and apical dendrites of CA1 PCs. (A–E) Low-magnification images of P-faces of a PC soma (A) and spiny apical dendrites from the proximal (B), middle (C) and distal (D) stratum radiatum and stratum lacunosum-moleculare (E) show a rather uniform immunogold labelling pattern. (A′–E′) High-magnification images of the boxed region in (A–E). (F) Immunogold particles for the Kv4.2 subunit are homogenously distributed around a branch point on a PC dendrite. (F′) High-magnification view of the boxed region in (F). dist. sr, distal stratum radiatum; mid. sr, middle stratum radiatum; prox. sr, proximal stratum radiatum; slm, stratum lacunosum-moleculare; sp, stratum pyramidale. Scale bars: 1 μm (A); 500 nm (B–F); 100 nm (A′–F′).

Electron microscopic analysis of the replicas revealed many gold particles labelling the Kv4.2 subunit on P-faces of somatic and dendritic plasma membranes, consistent with the intracellular location of the epitope (AA 454–469) recognized by the rabbit anti-Kv4.2 antibody. Gold particles were apparently randomly distributed and showed a rather similar distribution pattern in the plasma membranes of somata and main apical dendrites of CA1 PCs (Fig. 2B–E). Quantification of the immunogold reactions demonstrated a moderate distance-dependent increase in gold particle density along the proximo-distal axis of the main apical dendrites within the SR (proximal SR – 7.8 ± 3.8 gold/μm^2^; middle – 11.9 ± 3.7 gold/μm^2^; distal – 11.4 ± 2.8 gold/μm^2^; *n* = 5 rats; Table 1), with a slight decrease in dendrites of the SLM (8.9 ± 3.4 gold/μm^2^). The density of gold particles labelling the Kv4.2 subunit in the proximal apical dendrite was very similar to that found in the somata (6.9 ± 1.7 gold/μm^2^; Fig. 2A and A’). The average relative increase from the proximal to distal dendrites within the SR was 69 ± 50% in five rats.

**Fig. 3:**
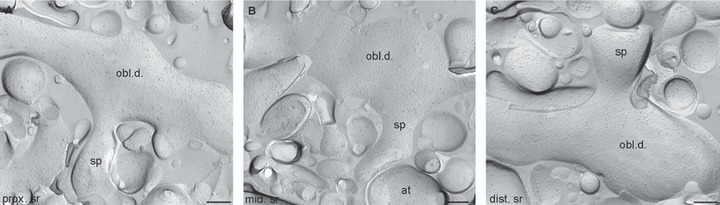
Distribution of immunogold particles for the Kv4.2 subunit in oblique dendrites and spines of CA1 PCs in the stratum radiatum. (A) Immunogold particles are homogenously distributed along the P-face of an oblique dendrite (obl.d.) in the proximal stratum radiatum (prox. sr). (B) Slightly higher immunogold particle density for the Kv4.2 subunit can be seen on an oblique dendrite from the middle stratum radiatum (mid. sr). Note that a spine (sp) with high density of immunogold particles emerges from the dendrite and faces an axon terminal (at) E-face. (C) A spiny oblique dendrite in the distal stratum radiatum (dist. sr) contains a large number of immunogold particles for the Kv4.2 subunit. Scale bars: 250 nm (A–C).

**Fig. 4:**
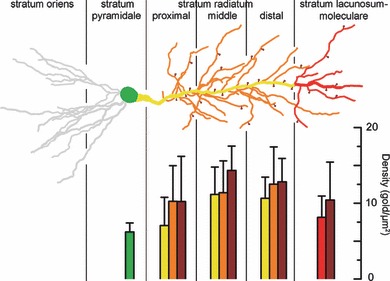
Densities of Kv4.2 immunogold particles in different somato-dendritic compartments of rat CA1 PCs. Bar graphs illustrate the background subtracted densities (mean ± SD, in gold/μm^2^) of immunogold particles in the somato-dendritic compartments. The bars are colour coded to different subcellular compartments as indicated in the schematic drawing of a CA1 PC. Note the moderate increase in the density along the proximo-distal axis of PCs within the stratum radiatum and the subsequent decrease in the stratum lacunosum-moleculare.

To investigate whether a much higher density of the Kv4.2 subunit in oblique dendrites or dendritic spines could mask the slight increase found in main apical dendrites and therefore result in a uniform staining of the SR as observed at the light microscopic level, we quantified gold particle densities in oblique dendrites and dendritic spines in the above-mentioned three subdivisions of the SR (Fig. 3). Gold particle densities in oblique dendrites (proximal SR – 11.0 ± 5.0 gold/μm^2^; middle – 12.1 ± 4.6 gold/μm^2^; distal – 13.3 ± 5.4 gold/μm^2^; *n* = 5 rats) and in dendritic spines (proximal SR – 11.0 ± 6.1 gold/μm^2^; middle – 15.1 ± 3.5 gold/μm^2^; distal – 13.6 ± 3.5 gold/μm^2^; SLM – 11.2 ± 5.7 gold/μm^2^; *n* = 5 rats; Table 1) showed an almost identical increasing–decreasing pattern to that found for the main apical trunks (Fig. 4). In each subregion of the SR, the average densities in oblique dendrites and spines were only 26 ± 16% higher than those found in the main apical trunks, demonstrating the lack of large quantitative differences in the densities of Kv4.2 subunit among these distinct dendritic compartments. The densities of gold particles in all examined compartments, but the somatic membranes, are above the non-specific labelling (background – 0.7 ± 0.7 gold/μm^2^; anova with Dunnett’s *post hoc* test, *P* < 0.05). Despite the increasing–decreasing tendency in the density of gold particles along the dendritic regions, statistical comparisons revealed no significant difference in the densities among these dendritic compartments (*P* = 0.08, anova). Previous studies suggested that A-type K^+^ channels potentially located around dendritic branch points have a critical role in gating the propagation of dendritic spikes towards the soma or into other dendrites (Cai *et al.*, 2004; Losonczy *et al.*, 2008). Therefore, we specifically investigated the distribution pattern and density of the immunogold particles for the Kv4.2 subunit on plasma membranes of dendritic branch points, but could not detect any clustering or increase in particle density (Fig. 2F and F’).

### Specificity of the Kv4.2 subunit immunogold labelling in CA1 PCs using SDS-FRL

**Fig. 5:**
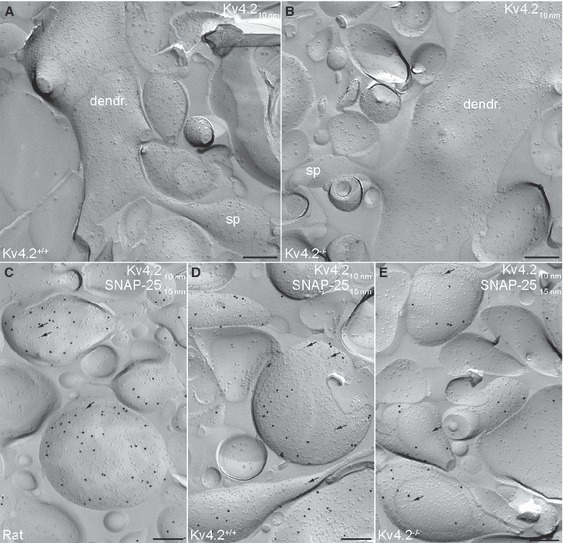
Specificity test for Kv4.2 subunit labelling on the axo-somato-dendritic surface of CA1 PCs using SDS-FRL. (A) Electron micrograph illustrating the P-face of a spiny CA1 PC dendrite of wild-type mouse immunolabelled for the Kv4.2 subunit. Note that the density and distribution pattern of the immunogold labelling for the Kv4.2 subunit in mouse is similar to that seen in rat. (B) The lack of immunogold labelling for the Kv4.2 subunit in a spiny dendrite of a Kv4.2^−/−^ mouse demonstrates the specificity of the labelling using SDS-FRL. (C) Axon terminals in rat identified by immunolabelling for SNAP-25 (15-nm gold) contain a low number of immunogold particles (arrows) for the Kv4.2 subunit (10-nm gold). (D and E) Immunogold particles for the Kv4.2 subunit were present in both Kv4.2^+/+^ (D) and Kv4.2^−/−^ mice (E) at approximately the same density. dendr, dendrite; sp, spine. Scale bars: 250 nm (A–E).

To validate the specificity of immunogold labelling on SDS-FRL, we repeated our reactions in control and Kv4.2^−/−^ mice (Fig. 5A and B). In control mice, the strength of the reaction was similar (*P* = 0.22, unpaired Student’s *t*-test) to that obtained in five rats, as assessed from the gold particle densities in the main apical dendrites in the middle of the SR (8.6 ± 2.6 gold/μm^2^, *n* = 3 control mice; Fig. 5A; Table 2), and showed a similar labelling pattern within the SR and SLM to that found in rats. In contrast, in Kv4.2^−/−^ mice, the mean density of gold particles on P-face structures (soma – 0.76 ± 0.48 gold/μm^2^; apical dendrite – 1.10 ± 1.17 gold/μm^2^; oblique dendrite – 1.40 ± 1.63 gold/μm^2^; SLM – 1.07 ± 0.88 gold/μm^2^; dendritic spine – 1.58 ± 1.61 gold/μm^2^; *n* = 3 mice; Fig. 5B; Table 3) was not significantly different from that obtained in the E-face structures around them (0.36 ± 0.06 gold/μm^2^; *n* = 3; *P* = 0.08, anova). The low insignificant labelling in Kv4.2^−/−^ tissue was consistent in all analysed somato-dendritic compartments throughout the depth of the SR and SLM (Table 3), demonstrating the specificity of immunogold labelling in all somato-dendritic compartments.

### The Kv4.2 subunit is excluded from the postsynaptic membrane specializations

**Fig. 6:**
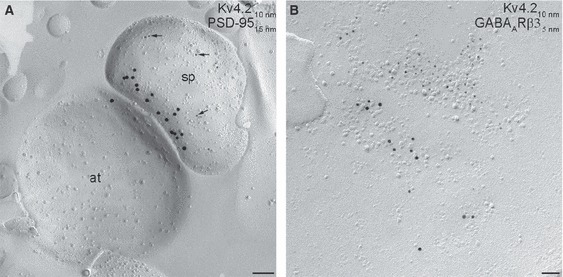
The Kv4.2 subunit is excluded from postsynaptic membrane specializations. (A) Electron micrograph illustrating an excitatory synapse, revealed by the accumulation of the postsynaptic density marker PSD-95 (15-nm gold). Arrowheads point to gold particles labelling the Kv4.2 subunit (10-nm gold) around the PSD. (B) High-magnification image of an inhibitory synapse identified by the enrichment of gold particles (5-nm gold) labelling the γ-aminobutyric acid (GABA)_A_ receptor β3 subunit. Note the clustering of gold particles labelling the Kv4.2 subunit (10-nm gold) in the extrasynaptic membrane, but not within the inhibitory synapse. Scale bars: 100 nm (A); 50 nm (B).

Next, we asked whether the Kv4.2 subunit is concentrated in GABAergic or glutamatergic synapses, as suggested to occur in the supraoptic nucleus (Alonso & Widmer, 1997), developing cerebellum (Shibasaki *et al.*, 2004), visual cortex (Burkhalter *et al.*, 2006) and subiculum (Jinno *et al.*, 2005). The SDS-FRL technique allows the visualization of neurotransmitter receptors and their associated proteins in both GABAergic (Kasugai *et al.*, 2010; Lorincz & Nusser, 2010) and glutamatergic synapses (Kulik *et al.*, 2006; Masugi-Tokita *et al.*, 2007). We colocalized the Kv4.2 subunit with PSD-95, which clearly marks the PSD of excitatory synapses on the P-face of the replica (Kulik *et al.*, 2006) and with the GABA_A_ receptor β3 subunit (Kasugai *et al.*, 2010; Lorincz & Nusser, 2010), which labels inhibitory synapses also on the P-face. We could not find any evidence for the enrichment of Kv4.2 subunits in GABAergic or glutamatergic synapses, only a few scattered gold particles were found around the periphery of some excitatory synapses (Fig. 6A). Immunogold particles labelling the Kv4.2 subunits infrequently clustered on somatic and dendritic membranes, but they never colocalized with the GABA_A_ receptor β3 subunit (Fig. 6B). These results provide evidence for the lack of synaptic accumulation of the Kv4.2 subunit in CA1 PCs.

### A low density of immunogold particles for the Kv4.2 subunit was found in SNAP-25-containing axon terminals, but it persisted in Kv4.2^−/−^ mice

Finally, we noticed that gold particles for the Kv4.2 subunit were not only confined to somato-dendritic plasma membranes, but were present in presumed presynaptic boutons at a low density. To unequivocally identify these weakly labelled structures, we performed double-labelling experiments for Kv4.2 and SNAP-25, a member of the SNARE complex present exclusively in axons (Hagiwara *et al.*, 2005). The strength of the Kv4.2 labelling in these double-labelling reactions in apical dendrites from the middle SR (10.9 ± 5.1 gold/μm^2^; *n* = 3 rats) was very similar to that found in single-labelling reactions (*P* = 0.77; unpaired Student’s *t*-test; Table 1). The SNAP-25-positive structures contained on average 2.4 ± 0.6 gold particles in each μm^2^ (*n* = 3 rats; Fig. 5C; Table 2). Although this density is only a quarter of that found on the somato-dendritic compartments, it is still significantly (*P* = 0.008, paired Student’s *t*-test) higher than the background labelling (0.5 ± 0.4 gold/μm^2^). The presence of significant immunolabelling in axon terminals is surprising, because the Kv4.2 subunit is conceived as a somato-dendritic ion channel. To confirm the specificity of axon terminal immunolabelling, we repeated these experiments in control and Kv4.2^−/−^ mice. The labelling intensity in control mice (2.6 ± 1.6 gold/μm^2^; *n* = 3 mice; Fig. 5D; Table 2) was very similar (*P* = 0.82, unpaired Student’s *t*-test) to that found in rats, but surprisingly the density of gold particles labelling the Kv4.2 subunit was almost identical in the axon terminals of control and Kv4.2^−/−^ mice (axon terminals – 2.2 ± 0.4 gold/μm^2^; *n* = 3 mice; *P* = 0.69, unpaired Student’s *t*-test; Fig. 5E; Table 2). This value was significantly (*P* = 0.004, paired Student’s *t*-test) higher than the background labelling on the surrounding E-face structures (0.6 ± 0.2 gold/μm^2^). Thus, our results reveal that the immunogold labelling in the somato-dendritic compartments is due to specific antibody–Kv4.2 subunit interactions, whereas the same antibody under identical experimental conditions provides a weak, non-specific labelling in axon terminals.

## Discussion

In the present study, we have applied a highly sensitive, high-resolution quantitative electron microscopic immunogold technique to investigate the densities of the Kv4.2 subunit in 13 distinct axo-somato-dendritic compartments of CA1 PCs. Our results revealed a previously unseen distribution pattern on the surface of PCs, where the density of Kv4.2 subunit shows a slight increase from proximal to distal dendrites in the SR, then a decrease in the SLM. The approximately 70% increase in the density within the SR is much less than the reported increase in I_A_ along the main apical trunk. Although the density of Kv4.2 subunit is higher in small-diameter oblique dendrites and dendritic spines than that in the corresponding main apical trunk, this moderate, approximately 25%, difference is unlikely to underlie a fundamentally distinct role of I_A_ in main vs oblique dendrites.

Our results demonstrating only a moderate distance-dependent increase in the density of the Kv4.2 subunit from proximal to distal dendrites in SR are in disagreement with results obtained with dendritic recordings of I_A_. Hoffman *et al.* (1997) have demonstrated a approximately sixfold increase in peak I_A_ within the same dendritic segments of CA1 PCs. What could be the reason for this discrepancy between I_A_ and Kv4.2 subunit, when dendritic I_A_ is exclusively mediated by this subunit? A possible explanation could be that Kv4.2 subunits are in distinct functional states along the apical dendrites of PCs; i.e. a large fraction of the channels in the proximal dendrites do not conduct K^+^ within a functionally relevant voltage range. We thus have to assume that certain posttranslational modifications or interactions with some auxiliary/associated proteins might regulate the function of these ion channels in a dramatic manner. The most widely studied posttranslational modulation of Kv4.2 channels is phosphorylation. It has been shown that Kv4.2 channels and I_A_ in hippocampal CA1 PC dendrites are regulated by protein kinase C (PKC), protein kinase A (PKA), CaMKII and mitogen-activated protein kinase (Hoffman & Johnston, 1998; Yuan *et al.*, 2002). Phosphorylation by PKC and PKA results in a 15-mV depolarizing shift in the activation curve of I_A_ in CA1 PC dendrites (Hoffman & Johnston, 1998). As a result, the same depolarization activates less I_A_ following PKA/PKC phosphorylation of the channels. Therefore, a distance-dependent decrease in the phosphorylation state of the Kv4.2 subunit along the proximo-distal dendrites of CA1 PCs could reconcile the differences in the activation curve of I_A_ in distal and proximal dendrites (Hoffman *et al.*, 1997), but could not explain the differences in the peak I_A_ density increase and that of Kv4.2 density in main apical trunks within the SR. It should also be noted that phosphorylation by PKA also causes internalization of Kv4.2 channels from spine plasma membranes of PCs (Kim *et al.*, 2007; Hammond *et al.*, 2008). Such regulation, however, results in changes in surface density of the channels, which is readily detectable with SDS-FRL. Alternatively, the function of Kv4.2 channels might be modulated by protein–protein interactions. Kv4 channels have been demonstrated to interact with K-channel-interacting proteins and dipeptidyl peptidase-like type II proteins, DPP6 and DPP10 (reviewed by Vacher *et al.*, 2008). It remains to be seen whether the interaction with these or other auxiliary proteins could completely silence Kv4.2 channels, and whether such proteins have an inverse distance-dependent distribution along the proximo-distal axes of PC dendrites.

Our high-resolution immunolocalization experiments revealed a novel, distance- and subcellular compartment-specific distribution pattern of an ion channel on the surface of CA1 PCs. Previously, our laboratory has examined the cell surface distribution of the hyperpolarization-activated mixed cation channel (HCN) subunit 1 in hippocampal, subicular and neocortical layer 5 PCs, and found an exclusive somato-dendritic distribution. It was reported that the density of HCN1 increases as a function of distance along the proximo-distal axis of the PC dendrite, with a steep, supralinear increase in the SLM (Lorincz *et al.*, 2002). Although the density of I_h_, mediated by HCN1/HCN2, and I_A_, mediated by the Kv4.2 subunit, is very similar, the subcellular distributions of the underlying channel subunits are remarkably different. Instead of having a monotonous increase, the density of the Kv4.2 subunit first increases slightly within the SR, then decreases in the SLM. Both of these distribution patterns are very different from that of the Nav1.6 subunit of the voltage-gated Na^+^ channels, which shows a distance-dependent decrease from proximal to distal dendrites of CA1 PCs (Lorincz & Nusser, 2010). Although our laboratory have carried out detailed quantitative electron microscopic analysis of the subcellular distribution of only these three voltage-gated ion channel subunits, light-microscopic immunolocalizations indicate that a large number of ion channel subunits (e.g. Kv1.1, Kv1.2, Kv2.1) have their unique cell surface distribution patterns within the same cell type (CA1 PCs). These data, taken together, prompt the hypothesis that each ion channel subunit has its unique subcellular distribution pattern on the surface of a given nerve cell.

Our results, showing the specificity of immunogold signal on the somato-dendritic compartments and the lack of it in axon terminals, demonstrate that a given antibody could provide specific and non-specific labelling in distinct subcellular compartments under identical experimental conditions. Although it seems like a minor technical issue, it has far reaching consequences in immunohistochemistry. It is also important to note that immunosignal obtained with light microscopic fluorescent techniques completely disappeared in Kv4.2^−/−^ mice, demonstrating that all immunosignals in these light microscopic reactions were due to specific antibody–Kv4.2 protein interactions. If we had taken these light microscopic control experiments as evidence for the specificity of our immunogold signal in SDS-FRL, we would have erroneously concluded the presynaptic presence of the Kv4.2 subunit. These results provide further evidence that the concept of ‘antibody specificity’ is erroneous. If the specificity was the ‘property’ of the primary antibody, then either all immunosignal under all experimental conditions should be specific or should be non-specific. This is clearly not the case. Watanabe *et al.* (1998) provided a compelling demonstration of this. When an antibody against the NR2A subunit was used under conventional conditions for light microscopic localization, the somatic and proximal apical dendritic cytoplasm of CA3 PCs became strongly labelled, a pattern that was identical in NR2A^−/−^ mice. However, when the tissue was treated with pepsin before the immunoreactions, the staining pattern changed to a punctate neuropil labelling, and the strong cytoplasmic labelling disappeared. This labelling pattern following antigen retrieval was completely absent in NR2A^−/−^ mice, demonstrating that the same primary antibody could provide specific and non-specific immunolabelling under different experimental conditions. Similar to this, the primary antibody used in the present study provided a strong cytoplasmic cell body staining for Burkhalter *et al.* (2006), which remained very similar in Kv4.2^−/−^ mouse tissue. The same primary antibody under our experimental conditions provided an exclusive neuropil labelling in the hippocampus and neocortex, which completely disappeared in Kv4.2^−/−^ mouse tissue, providing further evidence that the experimental conditions are crucial when the outcome of an immunoreaction is concerned. Furthermore, our SDS-FRL experiments demonstrated that even under identical reaction conditions, a primary antibody could provide both specific and non-specific labelling in different subcellular compartments. This is not surprising, because the cross-reacting molecule/protein could have a subcellular compartment-specific distribution pattern within the examined cell. Our results provide an alarming example, and emphasize that the specificity of immunosignal must be proven under each experimental condition in each brain region, cell type and even subcellular compartment (Lorincz & Nusser, 2008).

## Abbreviations

BSA: bovine serum albumin
GABA: γ-aminobutyric acid
HCN: hyperpolarization-activated mixed cation channel
I_A_: A-type K^+^ current
NGS: normal goat serum
PB: phosphate buffer
PC: pyramidal cell
PKA: protein kinase A
PKC: protein kinase C
PSD: postsynaptic density
SDS-FRL: sodium dodecyl sulphate-digested freeze-fracture replica-labelling
SLM: stratum lacunosum-moleculare
SP: stratum pyramidale
SR: stratum radiatum
TBS: Tris-buffered saline
